# Supplementary material for: Association of obesity, visceral adiposity, and sarcopenia with an increased risk of metabolic syndrome: A retrospective study
Source: PLoS One. 2021 Aug 17;16(8):e0256083. doi: 10.1371/journal.pone.0256083 (PMC8370618; doi:10.1371/journal.pone.0256083)
Supplement: Attachment — Submitted filename: Response to reviewers.docx [file pone.0256083.s002.docx]

**Response Letter**

Editors, PLOS ONE

Thank you for allowing the revision of our manuscript: ID PONE-D-21-15211 entitled “Association of obesity, visceral adiposity, and sarcopenia with an increased risk of metabolic syndrome: a retrospective study” We revised our manuscript in accordance with the reviewers’ suggestions. Our responses to the comments are as follows.

**Reviewer #1:**

The manuscript provides evidence of the association between body composition parameters (obesity, visceral adiposity, and sarcopenia) and metabolic syndrome in a large sample of subjects. The manuscript is technically sound and the data do support the conclusions however there are a few revisions or clarifications I would suggest the authors address.

**Comment 1>**
1. The authors indicated that data from 4,621 repeated checkups were excluded from the analyses it would have been interesting to assess how the body composition parameters may have predicted changes in MS status over time using the repeated visits from the 4,621 with repeat data as this data was available.

*Reply: Thank you for your valuable comments. It seems that causal relationship may be assessed by analyzing the data of subjects who underwent repeated health checkup. However, in this current study, we intended a cross-sectional study. We indeed have a plan to perform another study in the next step with the data of subjects who underwent repeated health checkup. As of now, it’s a pity that we can’t show you the repeat data, but we’ll be able to show you when the next paper is completed. We sincerely ask for your kind understanding.*

**Comment 2>**
2. The results are clear however, the authors should include the percentages for the total number of subjects (n=2238) with MS as well as the percentages for the total number of subjects who were in the obesity group, visceral adiposity group and sarcopenia group. I would further suggest either updating this in Table 1 and Table 2

*Reply: Thank you for your valuable comments. Based on your comments, we added the percentages of subjects with MS in Table 1. We also added the percentages of subjects in the* obesity group, visceral adiposity group and sarcopenia group in *Table 2.*

**Comment 3>**
3. Table 3 and the language describing table 3 is unclear, it states the prevalence of MS was significantly higher in those with obesity, visceral adiposity AND sarcopenia compared to those without however, the table appears to show these as mutually exclusive groups. That is to say, obesity vs none, visceral vs non, sarcopenia vs none NOT those with obesity, visceral adiposity AND sarcopenia I would assume the group of individuals whom may be categorized by all 3 conditions would be much smaller. Thus, I would suggest the authors use ‘or’ rather than ‘and’ to indicate these analyses were done separately for each of the 3 conditions.

*Reply: Thank you for your valuable comments. Based on your comments, we revised our manuscript. We used ‘or’ instead of ‘and’ to indicate these analyses were done separately for each of the 3 conditions.*

**Comment 4>**
4. It is unclear which models were used to assess the results reported in Table 3 and 4—were these done using chi-squared or the students t-tests? As these are counts for the number of parameters the authors should be more clear on how these results were modeled in the statistical analyses section.

*Reply: Thank you for your valuable comments. We added more specific contents in the statistical analyses section. In the results section, we revised some contents regarding Table 3 and Table 4.*

**Comment 5>**
5. Table 5 is clearly defined and easy to read as well as reported and described appropriately both in the analyses section as well as the results.

*Reply: Thank you for your valuable comments.*

**Reviewer #2:**

The authors have undertaken study to explore the association of multiple body composition parameters such as obesity, visceral adiposity and sarcopenia with metabolic syndrome. The sample size is large and study span of 5 year have resulted in robust data. Over all study represent a major portion of Asian population. Despite these merits the study is not novel and is just an extension of earlier study by Kim S H et al., “Association between sarcopenia level and metabolic syndrome” (PMID: 33739984).

**Comment 1>**

1. English should be improved throughout the text. Sentence needs to be rephrased and lines should be numbered throughout the manuscript.

*Reply: Thank you for your valuable comments. We rephrased some sentences and underwent additional English proofreading. And lines were numbered throughout the manuscript.*

**Comment 2>**
2. In discussion include a paragraph explaining the parameters that are included in the current study and merit of the current study over earlier study conducted by same author (PMID: 33739984).

*Reply: Thank you for your valuable comments. As you suggested, we added sentences explaining the parameters that are included in the current study and merit of the current study over earlier study.* *We revised our manuscript as follows.*

Discussion – 3^rd^ paragraph

The previous study of the same study population as this study (Figure 1) showed that sarcopenia diagnosed by BIA is independently associated with MS risk in a dose-response manner.^46^ Our previous study focused on the relationship between sarcopenia and MS.^46^ In contrast, the current study comprehensively analyzed the effects of obesity, visceral adiposity, and sarcopenia on the risk of MS. Our current study demonstrated that the risks for MS additively increased……

**Comment 3>**

3. The table 1 and table 2 are duplication of table 1 of previous published article (PMID: 33739984) by the same author represented in different way.

*Reply: Thank you for your valuable comments. Our current study and the previous published article share the same study population (N=13620), but the research topic and analysis methods were completely different between the two studies. However, since Table 1 and Table 2 in our current study have some overlap with the contents of the Table 1 of the previous paper, we added such contents in the main text and cited the previous paper in Table 1 and Table 2.*

**Comment 4>**
4. The figure 5B of the current study is duplication of figure 5 of previous published article (PMID: 33739984).

*Reply: Thank you for your valuable comments. Although the research topic and analysis methods were completely different between the two studies, patients who performed both CT and Inbody on the same day were the same. Therefore, the graph that correlated CT and Inbody in this current study was the same as the graph in the previous study. We added such contents in the main text and cited the previous paper in the Figure 5B.*

**Comment 5>**
5. Figure legends should be self-explanatory.

*Reply: Thank you for your valuable comments. Based on your comments, we revised the Figure legend of Figure 2.*

**Comment 6>**
6. The study is not novel.

*Reply:* Although this study is not novel, the sample size of our study is large and 5-year span resulted in robust data as you commented. And our data strongly support our conclusion. Thus, our study is thought to have sufficient merit. Thank you.

Thank you again for your insightful advice.

Yours sincerely,

Ji Bong Jeong, MD, PhD

Associate Professor

Department of Internal Medicine

Seoul Metropolitan Government Seoul National University Boramae Medical Center

20 Boramae-ro 5-gil, Dongjak-gu

Seoul 07061, Republic of Korea

Phone: +82-2-870-2222

Fax: +82-2-870-3863

E-mail: jibjeong@gmail.com
